# Giant Orbitoethmoidal Osteoma: When an Open Surgical Approach Is Required

**DOI:** 10.1155/2015/872038

**Published:** 2015-03-23

**Authors:** Hussam Abou Al-Shaar, Turki El Arjani, Michael S. Timms, Faisal Al-Otaibi

**Affiliations:** ^1^Division of Neurological Surgery, Neurosciences Department, King Faisal Specialist Hospital and Research Center, P.O. Box 3354, Riyadh 11211, Saudi Arabia; ^2^College of Medicine, Alfaisal University, Riyadh 11533, Saudi Arabia; ^3^Department of Otolaryngology, King Faisal Specialist Hospital and Research Center, P.O. Box 3354, Riyadh 11211, Saudi Arabia

## Abstract

Giant orbitoethmoidal osteoma in children is considered to be rare. This type of pathology can be associated with significant disfiguring proptosis and limitation of eye movement. Here, we report on a child who presented with a giant orbitoethmoidal osteoma that was removed through an orbitofrontal approach. The cosmetic result was excellent and evident immediately after surgery. A review of the literature complements this report.

## 1. Introduction

Osteomas are uncommon tumors that constitute 1% of all bone tumors and 11% of benign bone tumors [1]. Skull base osteomas arising in the ethmoid sinus are extremely rare, especially giant lesions [2–4]. Symptomatic lesions usually present with symptoms related to their anatomical location [3]. A computed tomography (CT) scan is the modality of choice to diagnose these lesions. Asymptomatic ethmoidal osteomas can be managed conservatively with serial radiological images, while symptomatic lesions should be managed surgically [3, 4]. Herein, we report on a child with giant orbitoethmoidal osteoma. In addition, a literature review of orbitoethmoidal osteomas is provided, with focus on surgical management options for giant orbitoethmoidal osteomas.

## 2. Case Report

**Figure 1:**
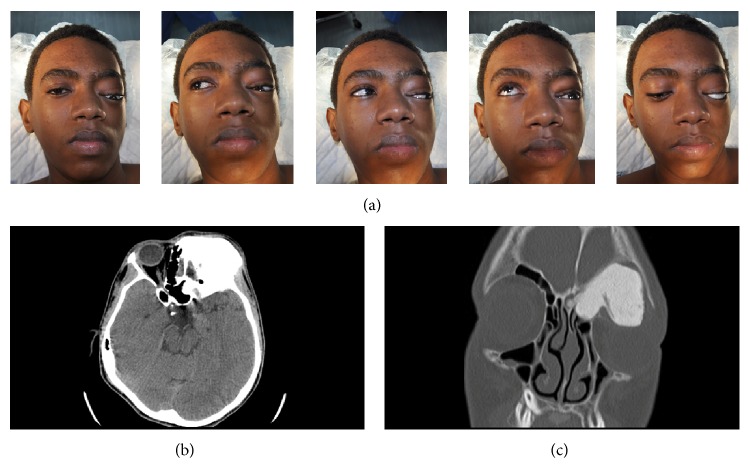
Patient photo demonstrating the degree of proptosis and limitation of eyemovements (a). Computerized tomography (axial (b) and coronal (c)) depicting giant osteoma feature at the orbitoethemoidal region and the degree of orbital compression.

A 16-year-old boy presented with progressive proptosis in the left eye that had first appeared 3 years earlier. He had recently developed limitation of left-eye movement associated with double vision. During physical examination, downward deviation and protrusion of the left eye and limitations of lateral and vertical gaze were evident (Figure 1). No papilledema or optic atrophy was detected on fundoscopic examination. A CT scan of the orbit revealed a hyperdense giant mass occupying the left supraorbital region, frontal sinus, and left ethmoid, with extension to the left nostril. In addition, the radiological feature of severe proptosis in the left eye was seen.

**Figure 2:**
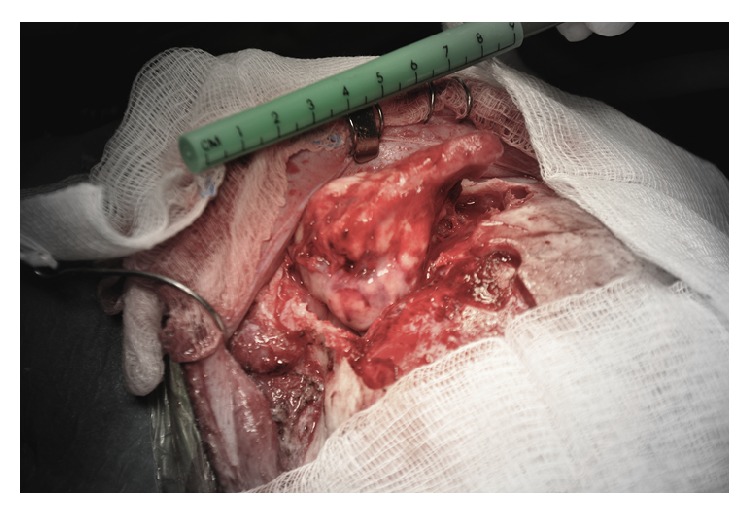
Intraoperative photo showing the large osteoma exposed through a fronto-orbital surgical approach.

**Figure 3:**
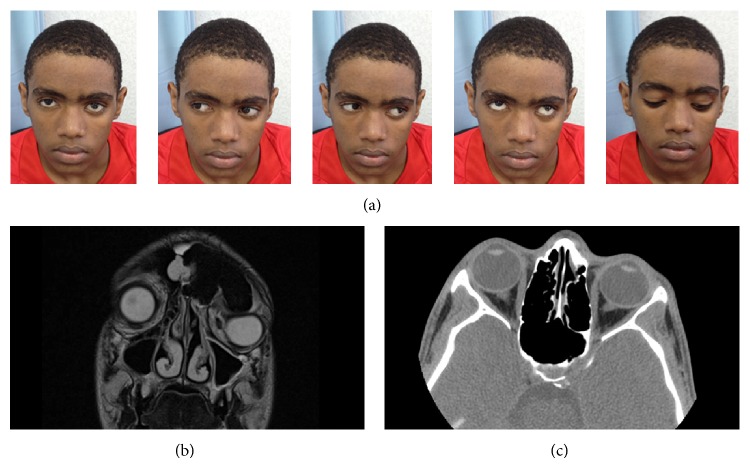
Postoperative patient photo demonstrating the resolution of proptosis and fulleye movements (a). Preoperative coronal MRI orbit showing the degree of compression on the orbit (b). Postoperative CT orbit showing the resolution of proptosis (c).

The features of this mass are suggestive of osteoma. The mass was removed through a left fronto-orbital approach. A supraorbital frontal craniotomy was carried out, exposing the osteoma at the supraorbital and the ethmoid regions (Figure 2). The supraorbital rim was involved by the mass. Reconstruction of the supraorbital rim and frontal bone with MEDPOR mesh was performed. The patient had an uneventful postoperative course. The cosmetic effect of the procedure was excellent immediately after surgery (Figure 3).

## 3. Discussion

Osteomas are exceedingly rare benign osteogenic tumors [3]. They can arise from any bony structure, with the paranasal sinuses being the most common location. They are most commonly encountered in the frontal sinus, followed by the ethmoidal, maxillary, and sphenoid sinuses, in that order [5]. The prevalence of osteomas is estimated to be 0.43–1% within the population [5, 6]. Osteomas larger than 3 cm in diameter and weighing more than 110 g are considered giant tumors [7]. Though most osteomas are sporadic, patients with Gardner's syndrome, an autosomal dominant disease, are at increased risk of developing osteomas alongside other diseases [5].

The etiology of osteomas remains elusive. Three different hypotheses have been described in the literature, identifying possible instigators for osteoma formation [2, 3]. The infectious theory is based on the presence of bony hyperplasia as a result of chronic infection and inflammation. The embryologic theory revolves around the persistence of embryologic or cartilaginous cell remnants in the junctional zone around the labyrinth of the ethmoidal bone. The traumatic theory describes the origin of osteomas in previous head injury sites, which might explain the slight increase in prevalence among males. A combination of the latter two theories remains the most largely accepted etiological theory.

Most osteoma patients are asymptomatic. It is estimated that around 1% of osteomas are discovered incidentally on sinus plain X-rays, while only 3% are detected via CT scans [8]. The close proximity of the orbit to the ethmoid sinus increases the risk of ocular symptoms of giant extending ethmoidal osteomas, as seen in our case. Although rare, proptosis, exophthalmos, diplopia, and other ocular symptoms may occur as a result of orbital displacement by the tumor and can be the first complaints in such patients [3, 9]. Surgical intervention is indicated in the presence of symptoms, significant growth, or extension beyond the sinus borders on follow-up imaging. Orbital and sphenoid sinus osteomas, however, regardless of their size or symptoms, should be resected whenever encountered because of the potential that these progressive, slow-growing tumors can compress the visual pathways and cause blindness [4].

Surgical techniques for the treatment of osteomas remain controversial. The surgical approach should be based on tumor size, tumor location, and the surgeon's preference and experience [3]. Symptomatic orbitoethmoidal osteomas have been managed surgically with open procedures for decades. The osteoplastic flap technique, anterior surgical exposure (craniofacial, transcoronal, and transcutaneous paranasal approaches), external fronto-ethmoidectomy, and lateral rhinotomy have all been described in the literature as possible techniques in the resection of giant osteomas that extend beyond the ethmoid sinus [4, 10–14]. The complications of the open surgical approaches are comparable with those encountered using the endoscopic technique. Recurrent frontal sinusitis, iatrogenic cranial nerve injury, visual disturbances, ptosis, CSF leakage, and postoperative hemorrhage have been reported in the literature with the open surgical techniques [15, 16].

With recent technological innovation in the surgical techniques and the introduction of endoscopic surgical interventions, many authors consider endoscopic resection of ethmoidal and orbitoethmoidal osteomas to be the new modality of choice for resecting such lesions [4, 17]. The minimally invasive endoscopic endonasal approach allows greater, closer, and more direct visualization of the tumor. Bony reconstruction of the walls, which is not usually indicated in ethmoidal osteomas, remains a limitation of the endoscopic approach [18]. In the presented case, the osteoma is extensive and involves the orbit with lateral extension making an endoscopic approach difficult. In addition, the superior orbital rim is involved, which mandates a reconstruction.

The surgical goal is to achieve complete surgical resection, protect vital structures (cribriform plate, optic nerves, lacrimal apparatus, anterior and posterior ethmoidal arteries, and the trochlea), and achieve favorable aesthetic outcomes [3]. Complete surgical excision can be achieved by en bloc resection of the osteoma whenever possible. Drilling the center of hard and/or giant osteomas to create a central cavity with thinning of the tumor peripheral walls, elevation of the tumor from the adjacent tissue and skull base, and subsequent removal with curette or by cutting should be considered for these lesions [7, 18]. In the presence of orbital extension, a similar approach can be considered for decompression of the orbit and optimal removal of the tumor [3]. Although extremely uncommon, incomplete resection of the osteoma is associated with increased risk of recurrence (up to 10%) [1, 5].

Most literatures describing giant orbitoethmoidal osteomas are case reports and small series. Therefore, the superiority of one surgical approach over the other is not yet established. However, in the presence of gigantic lesions with lateral extension beyond the orbital midline, as seen in our case, open surgical approaches are effective and were deemed more desirable.

## 4. Conclusion

Giant orbitoethmoidal osteomas have been managed with open approaches for decades. Only scarce case reports and small series are present in the literature. Our case adds to the growing literature on giant orbitoethmoidal osteomas managed successfully using open surgical approaches. Although the endoscopic endonasal approach demonstrates a safe and effective technique for the surgical management of giant orbitoethmoidal osteomas, the presence of a giant orbitoethmoidal osteoma that extends superolaterally into the orbital and frontal regions beyond the orbital midline might limit the endoscopic approach, and an open procedure offers the optimal surgical approach and orbitofrontal reconstruction.
